# Inhibition of salt inducible kinases reduces rhythmic HIV-1 replication and reactivation from latency

**DOI:** 10.1099/jgv.0.001877

**Published:** 2023-08-02

**Authors:** Helene Borrmann, Dini Ismed, Anna E. Kliszczak, Persephone Borrow, Sridhar Vasudevan, Aarti Jagannath, Xiaodong Zhuang, Jane A. McKeating

**Affiliations:** ^1^​ Nuffield Department of Clinical Medicine, University of Oxford, Oxford, UK; ^2^​ Department of Pharmacology, University of Oxford, Oxford, UK; ^3^​ Sleep and Circadian Neuroscience Institute (SCNi), Nuffield Department of Clinical Neurosciences, University of Oxford, Oxford, UK; ^4^​ Chinese Academy of Medical Sciences Oxford Institute, University of Oxford, Oxford, UK

**Keywords:** HIV-1, salt inducible kinases, circadian rhythms, host-pathogen interactions

## Abstract

Human immunodeficiency virus type 1 (HIV-1) causes a major burden on global health, and eradication of latent virus infection is one of the biggest challenges in the field. The circadian clock is an endogenous timing system that oscillates with a ~24 h period regulating multiple physiological processes and cellular functions, and we recently reported that the cell intrinsic clock regulates rhythmic HIV-1 replication. Salt inducible kinases (SIK) contribute to circadian regulatory networks, however, there is limited evidence for SIKs regulating HIV-1 infection. Here, we show that pharmacological inhibition of SIKs perturbed the cellular clock and reduced rhythmic HIV-1 replication in circadian synchronised cells. Further, SIK inhibitors or genetic silencing of *Sik* expression inhibited viral replication in primary cells and in a latency model, respectively. Overall, this study demonstrates a role for salt inducible kinases in regulating HIV-1 replication and latency reactivation, which can provide innovative routes to better understand and target latent HIV-1 infection.

## Introduction

HIV-1 is the causative agent of Acquired Immune Deficiency Syndrome (AIDS) and current therapies limit viral replication without eradicating infection [1]. HIV-1 infects immune cells, where HIV-1 RNA is reverse transcribed into DNA and integrates into the host cell genome. Once integrated, the virus can persist in reservoirs without actively replicating and with minimal detection by the immune system, a state called latency [2]. Pharmacological agents can reactivate latent HIV, which can be targeted by immune cells or anti-HIV drugs [3], however, most latency reversing agents have shown a modest impact on viral reservoirs [4]. Alternative therapeutic strategies to suppress the reactivation of latent genomes and to ‘block and lock’ the virus are being actively considered [5]. There is a need for studies to define host pathways that regulate HIV-1 transcription to direct novel therapeutic approaches. Recent reports demonstrate a role for endogenous 24 h oscillations, referred to as circadian rhythms, in viral infection [6–8] and immune responses [9, 10]. Cell-associated HIV RNA in peripheral blood CD4+ T cells of people living with HIV (PLWH) receiving antiretroviral therapy show a diurnal variation [11] and associate with the expression of the key circadian transcription factor brain and muscle ARNT-like 1 (BMAL1) [12]. We previously published that pharmacological modulation of key clock factors alters HIV-1 infection [13] and that the cell intrinsic clock regulates rhythmic HIV-1 replication [14], highlighting a role for circadian pathways in regulating viral transcription.

At a molecular level, each cell has an intrinsic clock machinery, which is orchestrated by interlinked transcriptional/translational feedback loops [15]. Beyond the regulation through this core circadian machinery, other protein families have been identified that contribute to circadian regulation, among others salt inducible kinases (SIKs) which consist of three members: SIK1, SIK2, and SIK3 [16]. SIKs convey signals through phosphorylation of multiple targets, the best understood of which are class IIa histone deacetylases (HDACs) and cAMP-regulated transcriptional coactivators (CRTCs) [17, 18]. The CRTC1-SIK1 pathway regulates entrainment of circadian rhythms by suppressing the effects of light on clock [19] and SIK3 was shown to destabilise the core circadian factor Period 2 (PER2) [20]. Various kinases have been reported to regulate HIV-1 infection [21, 22], however, the interplay of SIKs and HIV-1 has not been studied to date. We hypothesised that SIKs regulate rhythmic HIV-1 replication and latency reactivation and tested this by pharmacological inhibition, as well as transient siRNA silencing of Sik expression *in vitro*. We found that SIK inhibition perturbed the cellular clock, reduced HIV-1 replication and decreased the reactivation of latent genomes. Overall, this study provides fundamental insights into the regulation of HIV-1 by SIKs and offers a new perspective on viral latency.

## Methods

### Cell lines and primary cells

HEK293T cells and human bone osteosarcoma epithelial cells (U-2 OS) were obtained from ATCC and cultured in Gibco DMEM medium (high glucose, GlutaMAX supplement, pyruvate; Life Technologies) containing 10 % FBS and 1 % penicillin/streptomycin (Life Technologies). pABpuro-BlucF plasmid (Addgene #46824) was used to generate U-2 OS cells stably expressing the *Bmal1* promoter upstream of luciferase (Bmal1-luc) and cells maintained in 2 µg ml−1 puromycin (Life Technologies). J-Lat cells (clone 6.3) were provided by Professor Xiaoning Xu (Imperial College, London) and maintained in RPMI-1640 medium (Life Technologies) containing 10 % FBS and 1 % penicillin/streptomycin (Life Technologies).

PBMCs were isolated from leukapheresis cones purchased from NHS Blood and Transplant (Oxford, UK), with ethical approval for research use and written informed consent from all donors. CD8+ T cells were depleted (using a CD8 Micro Bead kit from Miltenyi Biotec) and cells cultured in RPMI-1640 (Life Technologies) containing 10 % FBS, 1 % penicillin/streptomycin (Life Technologies), 1 % sodium pyruvate (Sigma), 1 % Glutamax (Life Technologies), 1 % non-essential aminoacids (Life Technologies) and 2 mM beta-mercaptoethanol (Life Technologies). Cells were activated with 50 IU ml−1 IL-2 (Proleukin; Novartis), 0.01 µg ml−1 soluble human anti-CD3 (R and D; clone UCHT1) and 0.1 µg ml−1 soluble human anti-CD28 (Life Technologies; clone CD28.2) for 3 days before infection. All work with primary human cells was compliant with institutional guidelines. The first three biological repeats employed cells from single donors, whilst repeats four and five employed cells pooled from three donors.

### Reagents

SIK inhibitors HG-9-91-01, YKL-05-099 and ARN-3236 (Cambridge Bioscience Ltd) were dissolved in dimethyl sulfoxide (Life Technologies). Cellular cytotoxicity was determined using a lactate dehydrogenase (LDH) assay (Promega). Silencing RNAs for Sik1, Sik2 and Sik3, and siRNA controls were obtained from Thermo Fisher. LIVE/Dead Fixable Aqua for flow-cytometry was purchased from Life Technologies.

### Primers

Oligonucleotide sequences (Life Technologies).

**Table:** 

	Forward 5’ – 3’	Reverse 5’ – 3’
B2M (human)	CTACACTGAATTCACCCCCACTG	ACCTCCATGATGCTGCTTACATG
Sik1 (human)	GATGCCACCAAAGCAGCTACAG	CCTGCGTGAAATCCACAGTCTTG
Sik2 (human)	GAGCAGGTGAAAGTGCAGATCG	TGTAGCGGTCCATGCACATGGC
Sik3 (human)	CGAAGTTTGTGAACTGGCAGGTG	AAGGCGTGATGCTCTGGAGGTA
NL4.3 Gag	CGAGAGCGTCGGTATTAAGC	CTGAAGGGATGGTTGTAGCTG

### Generation of viral stocks

HEK293T cells were transfected with plasmids encoding NL4.3 R-E-luc (provided by the NIBSC AIDS Repository) and the VSV-G expression plasmid as previously reported [23] using polyethylenimine (PEI, Polysciences). Medium was replaced 4 h post-transfection with DMEM medium without antibiotics supplemented with 10 mM HEPES and virus was harvested 48 h later. Viral stocks were quantified by measuring the reverse transcriptase (RT) activity using a qPCR-based product-enhanced RT (PERT) assay [24].

### Time course experiments

U-2 OS cells were infected with VSV-G-pseudotyped HIV-1 NL4.3-luc (100 U RT/ 10^6^ cells) for 24 h. Infected cells, or U-2 OS stably expressing Bmal1-luc were synchronised by serum shock with 50 % FBS for 1 h, and 24 h post-serum shock medium was changed to DMEM lacking Phenol Red (Life Technologies), supplemented with 100 µM luciferin (VivoGlo, Promega) and each drug. Luciferase activity was recorded at 30 min intervals for 48 h using a CLARIOstar luminometer (BMG Labtech), where cells were kept at 37 °C and 5 % CO2. Cycling datasets were detrended with BioDare2 [25], normalised to control peak expression and curves fitted using Prism 9 (GraphPad). It is important to note that this normalisation sets the baseline of all data to zero. Representative raw data for each experiment can be found in Fig. S1.

### HIV-1 infection experiments

U-2 OS cells or activated CD8 depleted PBMCs were infected with NL4.3-luc VSV-G (100 U RT/ 10^6^ cells) for 24 h. Cells were washed and medium supplemented with drugs at indicated concentrations for 24 h, followed by detection of luminescence using a Firefly Luciferase Assay kit (Promega) and a Glomax luminometer (Promega).

### Flow cytometry

J-Lat cells were incubated with different doses of ARN-3236 in combination with 100 ng µl−1 TNFα for 24 h. Cells were stained with LIVE/Dead Fixable Aqua (Life Technologies) and fixed with 4 % paraformaldehyde (PFA) (Santa Cruz) for 10 min at room temperature. Data were acquired using an Attune NxTflow cytometer (Thermo Fisher) and analysed using FlowJo (TreeStar).

### siRNA mediated silencing

Sik1, Sik2 and Sik3 siRNA or a scrambled control were transfected into J-Lat cells using Fugene SI (Promega). At 48 h post-transfection cells were stimulated with 100 ng µl−1 TNFα for 24 h, followed by lysis and gene expression analysis of Sik1, 2 and 3 or HIV Gag by qPCR.

### RT-qPCR

Cells were lysed and RNA extracted using the RNeasy kit from QIAGEN. Residual DNA was digested (TURBO DNase free kit, Thermo Fisher) and equal amounts of RNA were used for cDNA synthesis (cDNA Synthesis Kit, PCR Biosystems). qPCR was performed using Fast SYBR Master Mix (PCR Biosystems) in a LightCycler96 (Roche) with the primers listed above. mRNA expression was calculated relative to Beta-2-Microglobulin (Β2M) expression using the ΔCt method.

### Statistical analysis

*P-*values were determined using Mann-Whitney or Kruskal-Wallis tests using Prism 9 (GraphPad). In the figures * denotes *P*<0.05, **P<0.01, ***P<0.001, all data are presented as mean values±SEM.

## Results and discussion

**Fig. 1.**
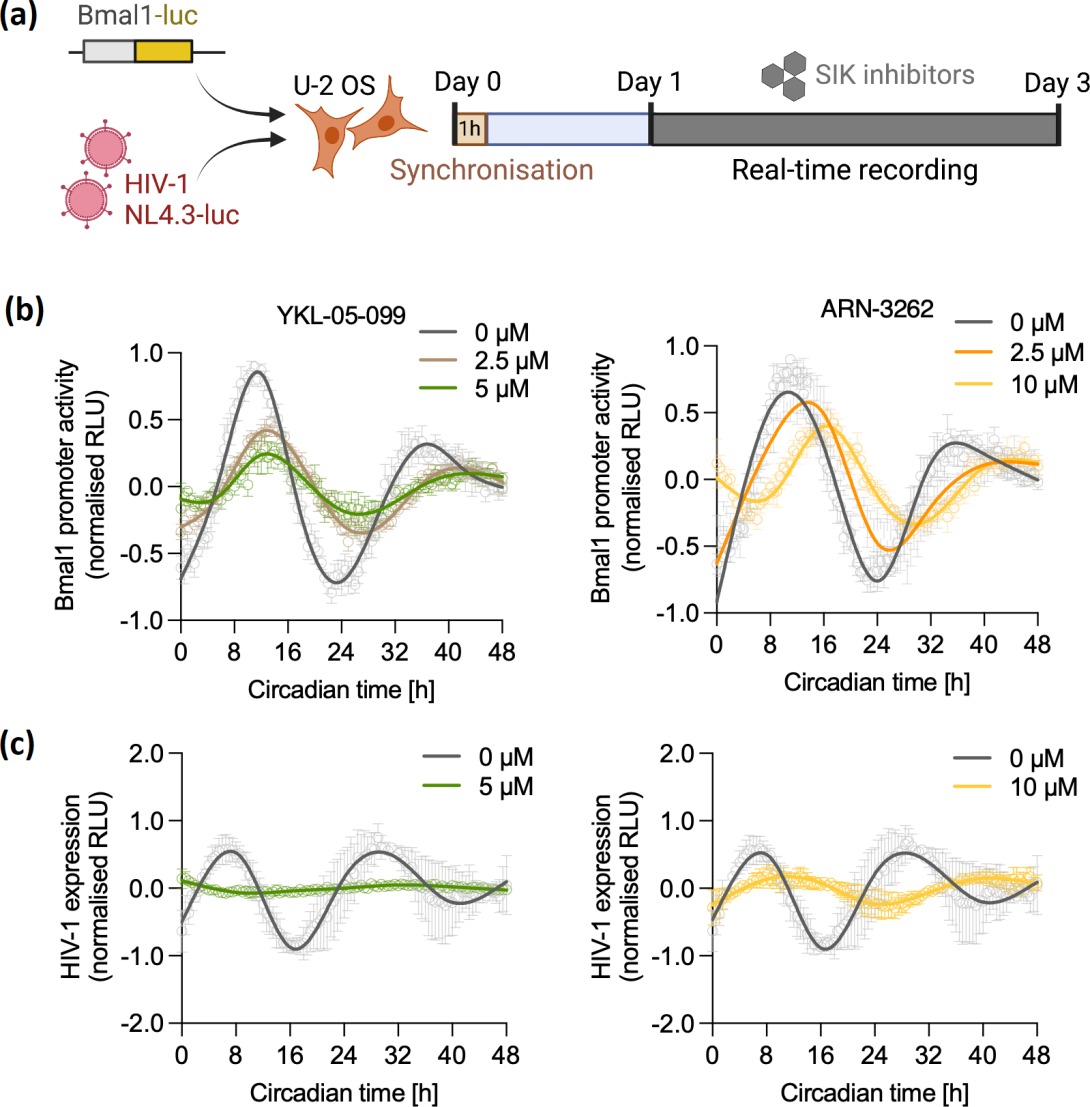
Salt inducible kinase inhibition dampens rhythmicity of Bmal1 promoter activity and HIV-1 replication. (**a**) Experimental protocol: U-2 OS cells stably expressing the Bmal1 promoter driving luciferase expression (Bmal1-luc) or infected with HIV NL4.3 R-E-luc (NL4.3-luc) VSV-G were synchronised by serum shock for 1 h. 24 h later, cells were treated with SIK inhibitors and luciferase activity (Relative Light Units, RLU) recorded every 30 min for 48 h. (**b**) Synchronised U-2 OS Bmal1-luc cells were treated with SIK inhibitors YKL-05-099 or ARN-3236, and luciferase measured as readout of Bmal1 promoter activity (mean±S.E.M., *n*=3, normalised to peak). (**c**) U-2 OS cells were infected with HIV NL4.3-luc VSV-G for 24 h, synchronised and treated with SIK inhibitors YKL-05-099 or ARN-3236. HIV-1 expression was measured by monitoring luciferase expression (mean±S.E.M., *n*=3, normalised to peak). Raw data in Fig. S1.

**Fig. 2.**
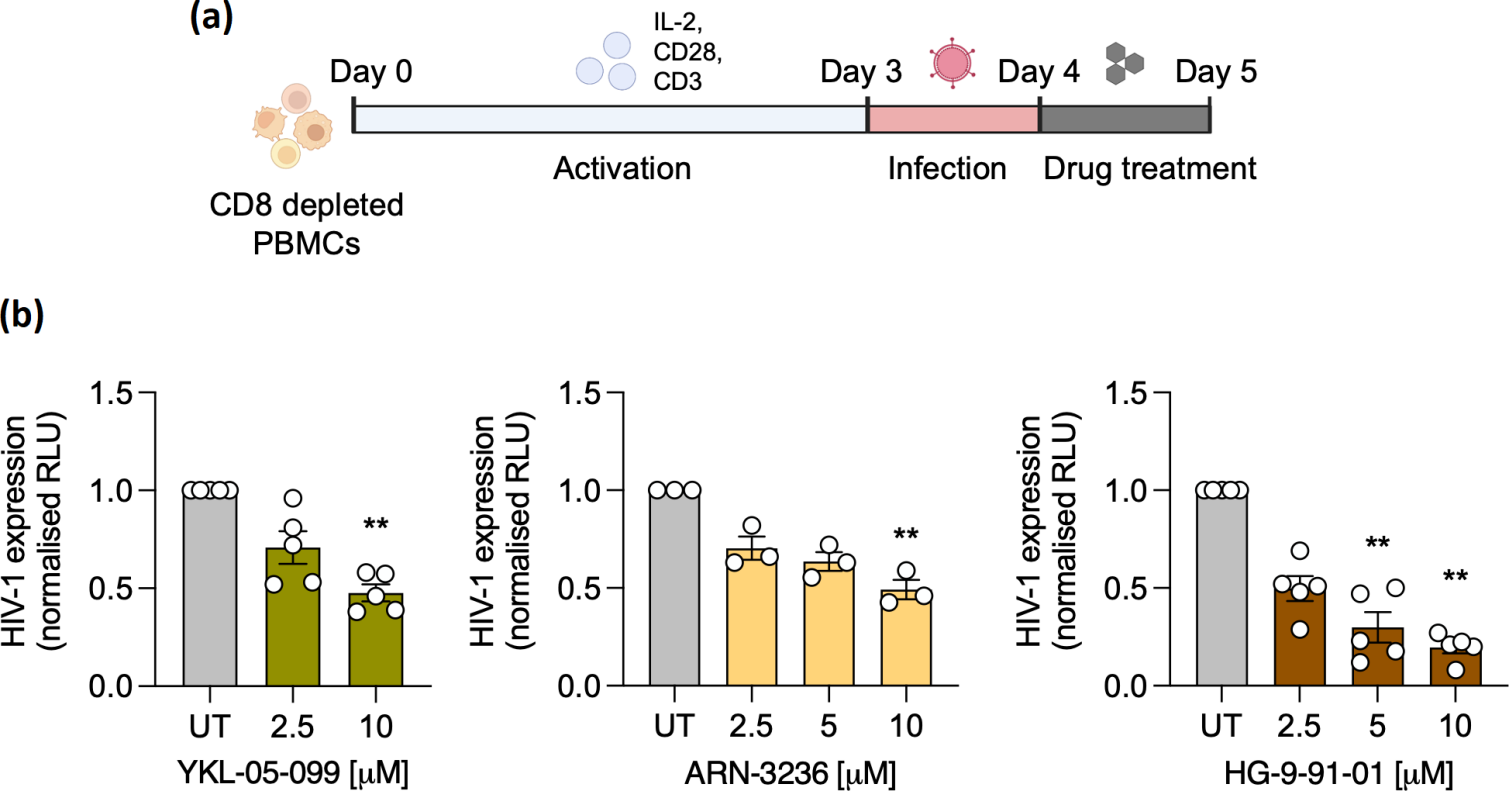
Pharmacological inhibition of salt inducible kinases reduces HIV-1 replication in primary T cells. (**a**) Experimental protocol: peripheral blood mononuclear cells (PBMCs) were isolated, CD8+ T cells depleted and cells stimulated with IL-2, anti-CD3 and anti-CD28 for 3 days. Cells were infected with HIV NL4.3-luc VSV-G for 24 h and treated with drugs for 24 h. (**b**) Activated CD8 depleted PBMCs infected with NL4.3-luc VSV-G were treated with different doses of YKL-05-099, ARN-3236 or HG-9-91-01, cells lysed and luciferase measured as readout for HIV-1 expression (mean±S.E.M., *n*=3–5, Kruskal-Wallis ANOVA). Data is shown relative to control untreated (UT) cells. The first three biological repeats employed cells from single donors, whilst repeats four and five employed cells pooled from three donors. **Denotes *P*<0.01.

A number of reports demonstrate that pharmacological inhibition of SIKs can modulate circadian rhythmicity and sleep homeostasis. SIK1 inhibition induced a phase-shift of circadian rhythms in fibroblasts using indirubin-3-monoxime (I3M) [19], and the pan-SIK inhibitor HG-9-91-01 modulated phosphorylation of synaptic phosphoproteins important for sleep homeostasis in mice [26]. We evaluated YKL-05-099 (a more selective analogue of HG-9-91-01) and a structurally unrelated SIK inhibitor ARN-3236 [27] for their ability to modulate cellular circadian rhythms using human bone osteosarcoma epithelial cells (U-2 OS), a well-established circadian *in vitro* model which shows robust rhythmic expression of circadian genes [14, 28]. U-2 OS cells that stably express the *Bmal1* promoter driving expression of luciferase were synchronsed by serum shock [29], followed by real-time recording of promoter activity in the presence of SIK inhibiting compounds 24 h post-synchronsation (Fig. 1a). We observed a dampening in amplitude and phase shift of *Bmal1* promoter cycling (Figs 1b and S1a, available in the online version of this article) demonstrating a perturbation of the cellular clock. Next, prior to serum shock synchronsation, U-2 OS cells were infected with the HIV-1 reporter virus NL4.3 R-E-luc (NL4.3-luc) that can undergo a single cycle of replication and was complemented with vesicular stomatitis virus encoded G protein (VSV-G) as previously described [14]. Viral activity was monitored real-time in the presence of YKL-05-099 or ARN-3236 and both treatments inhibited rhythmic HIV-1 replication (Figs 1c and S1b), in the absence of cytotoxicity (Fig. S2). Since U-2 OS cells do not reflect the natural reservoir of HIV *in vivo,* we extended these results to peripheral blood mononuclear cells (PBMCs) depleted of CD8+ cells to enrich CD4+ T cells, which are a key cellular site of HIV-1 replication. We observed a dose dependent reduction of HIV-1 reporter virus activity by measuring luciferase following treatment with several SIK inhibitors, YKL-05-099, ARN-3236 and HG-9-91-01 (Fig. 2a, b). Consistently, HIV-1 Gag RNA levels and Bmal1 transcripts were reduced in these cells (Fig. S3a, b), demonstrating a role for these compounds to limit active HIV-1 replication.

**Fig. 3.**
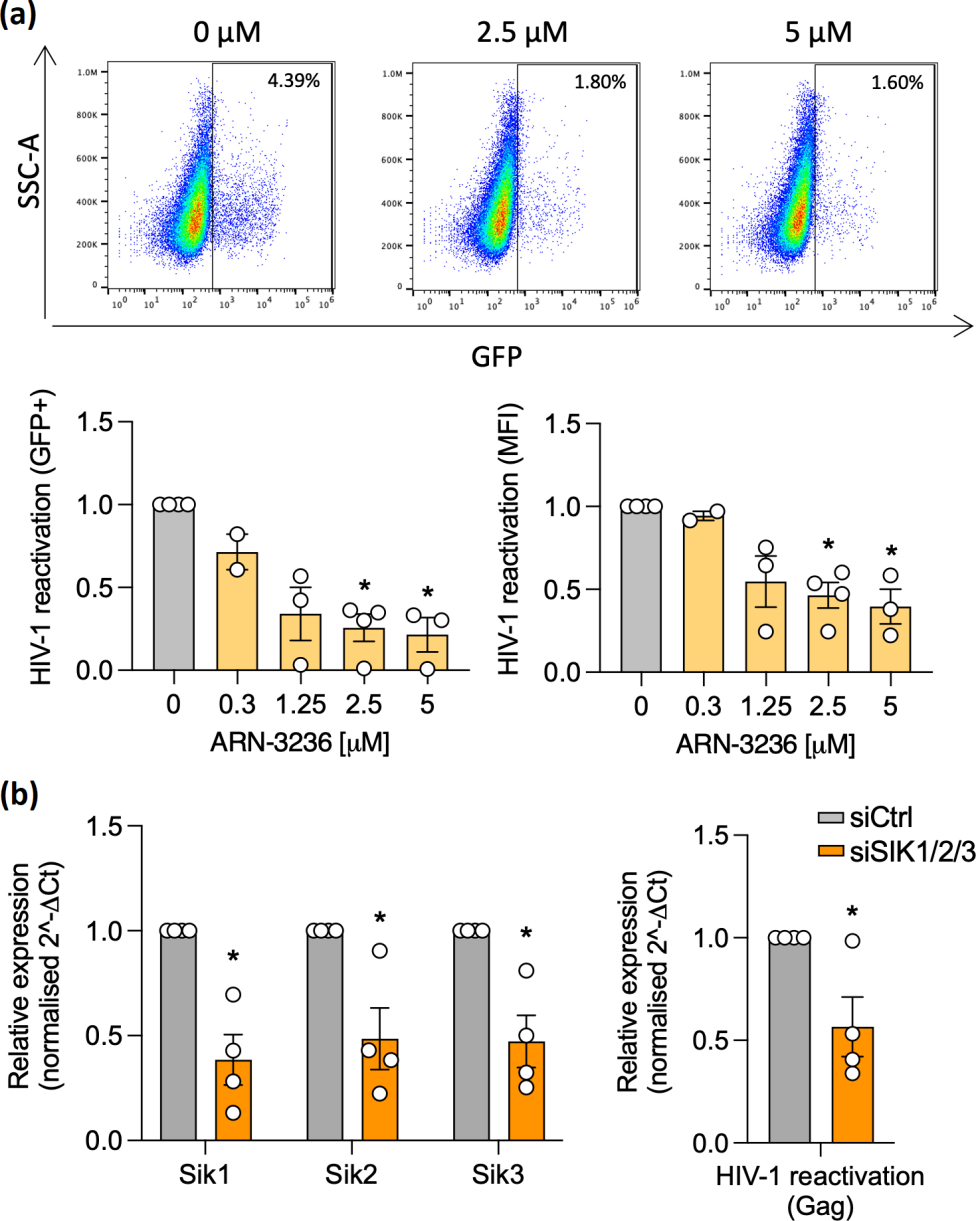
ARN-3236 treatment or knock-down of SIK expression reduced the reactivation of latent HIV in J-Lat cells. (**a**) J-Lat T cells were treated with different doses of ARN-3236 in combination with TNFα (100 ng µl^−1^) for 24 h. HIV-1 reactivation was measured by analysing the percentage of GFP+ cells and their mean fluorescence intensity (MFI) by flow cytometry. Representative dot plots are shown for ARN-3236 (2.5 µM and 5 µM) and data plotted relative to control (mean±S.E.M., *n*=2–4, Kruskal-Wallis ANOVA). (**b**) J-Lat cells were transfected with a pool of siRNAs targeting Sik1, 2 and 3 or scrambled control (siCtrl). Two days post-transfection cells were activated with TNFα (100 ng µl^−1^) for 24 h, followed by lysis and gene expression analysis of Sik1, Sik2 and Sik3 or HIV Gag RNA by qPCR (mean±S.E.M., *n*=4, Mann-Whitney test). Data is shown relative to control. *Denotes *P*<0.05, **P<0.01.

Intrigued by the anti-viral phenotype, we hypothesised that SIK inhibitors could limit the reactivation of latent HIV-1. We tested the most specific compound, ARN-3236 [27], for its effects on latency reactivation in J-Lat cells (clone 6.3), a T cell line which harbours a transcriptionally silent integrated copy of HIV-1 genome that encodes green fluorescent protein (GFP) [30] and is widely used in latency-altering drug screens [31]. Treating J-Lat cells with tumour necrosis factor alpha (TNFα) activates viral transcription and GFP expression. Combined treatment of J-Lat cells with ARN-3236 and TNFα showed a dose-dependent reduction in the percentage and mean fluorescence intensity (MFI) of GFP expressing cells (Figs 3a and S4), suggesting a role for SIKs in regulating HIV transcription. The frequency of reactivated cells (~5 %) is lower than earlier studies reported by ourselves [32] and others [30], but the inhibitory phenotype was consistent across multiple biological repeats. As many small molecule inhibitors can show off-target effects and SIK inhibitors have been reported to modulate the activity of other kinases [27], we complemented these findings with a genetic approach. We silenced all Sik isoforms in J-Lat cells using a pool of siRNAs, treated cells with TNFα and extracted RNA to confirm the knock-down and measure HIV-1 RNA by qPCR (Fig. 3b). We quantified HIV-1 group specific antigen (Gag) RNA levels which is the major structural HIV-1 protein and indicative of functional HIV-1 transcription initiation [33, 34]. Reassuringly, we observed a reduction in the abundance of HIV-1 RNA (Fig. 3b), supporting a pro-viral role for SIKs in regulating viral transcription.

A previous report identified Sik1 in an ingenuity pathway analysis of kinases regulating HIV-1, however, it was not studied further [21]. Our experimental study illustrates that SIK inhibitors perturb the cellular circadian clock, reduce rhythmic HIV-1 replication and inhibit viral replication in primary T cells. Pharmacological and genetic disruption of SIKs reduced TNFα mediated reactivation of HIV-1 in the J-Lat T cell model, altogether highlighting a pro-viral role for SIKs in regulating HIV-1 infection. SIKs phosphorylate CRTC causing its retention in the cytoplasm, and SIK inhibitors initiate CRTC translocation into the nucleus where it exerts its function. A recent report demonstrated that CRTC2 suppressed HIV-1 transcription by preventing RNA polymerase II from binding the HIV-1 long terminal repeat (LTR) [35]. This could point towards a mechanism whereby SIK inhibitors lead to CRTC2 mediated reduction of RNA polymerase II binding to the HIV-LTR, causing the anti-viral phenotype. However, further studies, like chromatin immunoprecipitation of RNA polymerase II, would be needed to validate this hypothesis.

Additionally, SIK inhibition leads to increased nuclear localisation and activity of class IIa HDAC [17]. HDAC inhibitors have been extensively studied for HIV latency reactivation as they increase accessibility of integrated viral genomes to bind transcription factors and to activate viral transcription [36]. Many treatment regimens target a broad range of HDAC classes [36], however, primarily class I HDACs are recruited to the HIV-1 LTR and cause chromatin remodelling through histone deacetylation [37]. It is currently thought that class IIa HDACs have little or no deacetylase activity [38], making class I specific HDAC inhibitors more effective [37, 39]. Importantly, class IIa HDACs act as adaptors by recruiting class I HDACs and other transcription factors, thereby contributing to transcriptional regulation [38]. Since SIK inhibitors increase HDAC class IIa localisation in the nucleus, these could recruit HDAC class I proteins that repress HIV-1 transcriptional activity. This speculative mechanism requires experimental validation and could contribute to greater understanding of the complex regulation of HIV-1 infection by HDACs.

Aside from CRTC and HDACs, SIKs are involved in a range of pathways regulating various cellular processes, e.g. metabolism [40] and inflammation [41], which could influence HIV-1 infection. It remains unclear whether SIKs directly regulate HIV-1 replication or whether their activity is mediated through the circadian clock or other pathways and we hope this short report stimulates further studies in this area. It would be interesting to elucidate the underlying mechanisms and clinical importance, for instance, by testing the salt inducible kinase inhibitors for their potential to limit viral reactivation in *ex vivo* CD4+ T cells from PLWH on ART. Moreover, using independent latent cell clones and reactivation stimuli would help assess the breadth of SIK inhibition on HIV-1 reactivation. ARN-3236 is in Phase I clinical trials for ovarian, peritoneal and other solid tumours and several SIK inhibitors are under development for clinical use [16], suggesting their potential for therapeutic applications in the future. In summary, our study highlights the importance of SIKs for HIV-1 replication and latency reactivation, and will guide future studies to dissect the mechanistic interplay. Ultimately, this could inform and complement existing therapeutic approaches.

## Supplementary Data

Supplementary material 1
