# Supplementary material for: Inhibition of salt inducible kinases reduces rhythmic HIV-1 replication and reactivation from latency
Source: J Gen Virol. 2023 Aug 2;104(8):001877. doi: 10.1099/jgv.0.001877 (PMC10721046; doi:10.1099/jgv.0.001877)
Supplement: Supplementary material 1 [file jgv-104-1877-s001.pdf]

Supplemental information

Inhibition of salt inducible kinases reduces rhythmic HIV-1 replication and reactivation from latency.

Helene Borrmann, Dini Ismed, Anna E. Kliszczak, Persephone Borrow, Sridhar Vasudevan, Aarti Jagannath, Xiaodong Zhuang, Jane A. McKeating

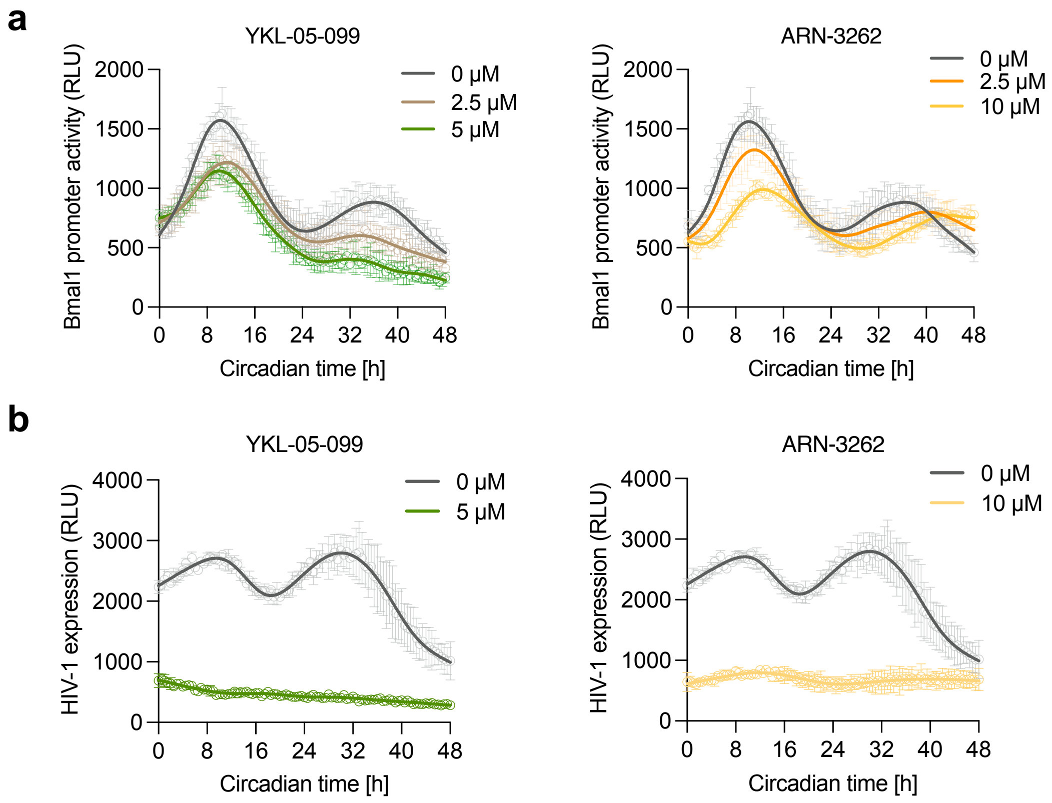

Supplementary Figure 1. Raw luminescence values for real time measurements. (a) U-2 OS cells stably expressing Bmal1 promoter-driven luciferase were synchronised, treated with different concentrations YKL-05-099 or ARN-3236 and luciferase activity measured at 30 min intervals starting 24 h post synchronisation (representative of n=3, mean ± S.D., related to Figure 1b). **(b)** U-2 OS cells were infected with HIV NL4.3-luc VSV-G, synchronised by serum shock, treated with YKL-05-099 or ARN-3236, and luciferase measured as readout for HIV-1 expression (representative of n=3, mean ± S.D., related to Figure 1c).

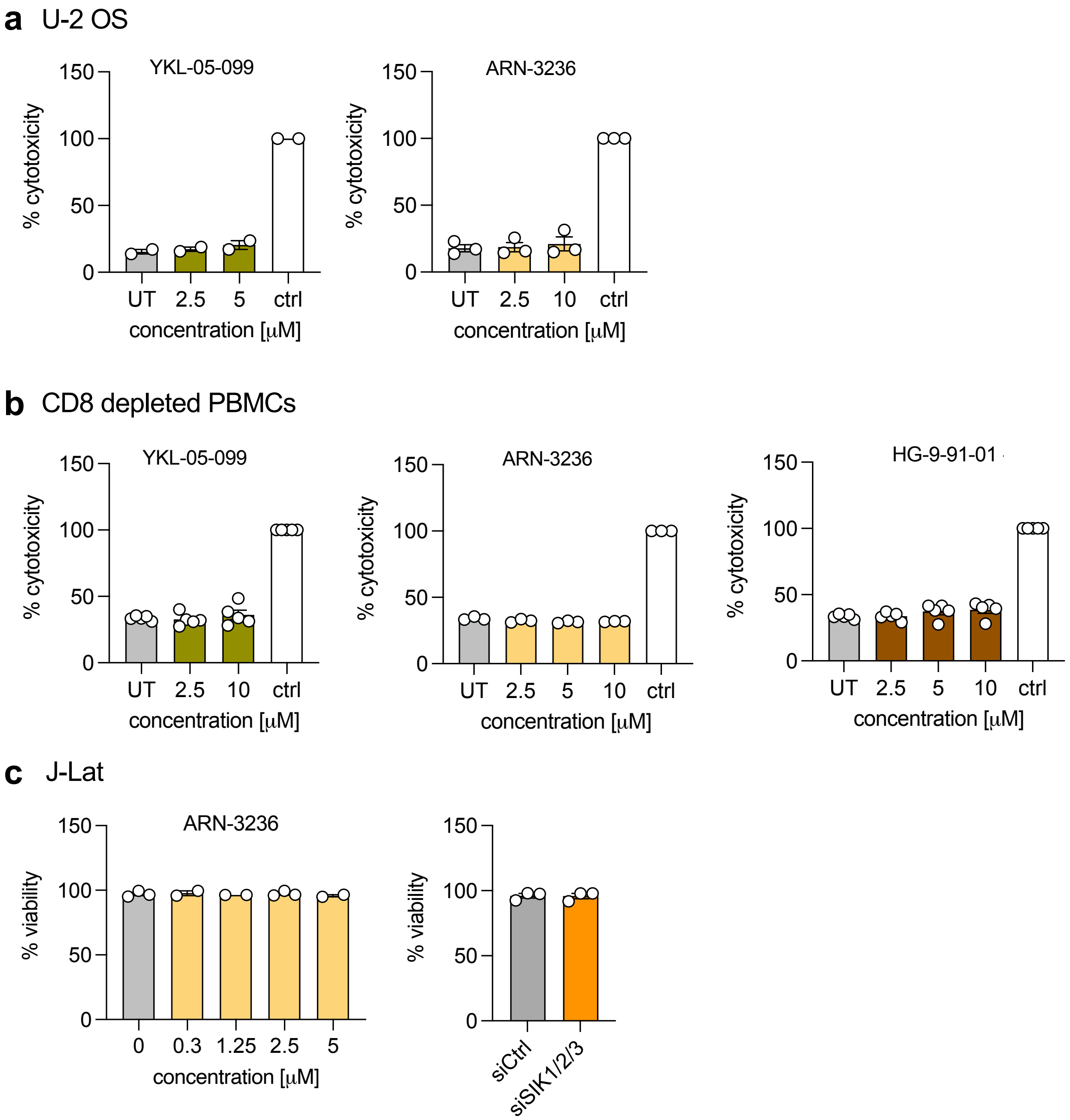

Supplementary Figure 2. Non-cytotoxicity of SIK inhibitors for various cell types. (a) U-2 OS cells or **(b)** activated CD8 depleted PBMCs were infected with HIV NL4.3-luc VSV-G, treated with SIK inhibitors YKL-05-099, ARN-3236 or HG-9-91-01 at a range of doses for 24 h and cytotoxicity determined using an LDH assay (mean ± S.E.M., n = 2-5). Data are expressed relative to the positive control representing total cell lysate (100% cytotoxicity). The first three biological repeats with CD8 depleted PBMCs employed cells from single donors, whilst repeats four and five employed cells pooled from three donors. **(c)** J-Lat cells were treated with TNFα alone or in combination with different doses of ARN-3236 for 24 h, or J-Lat cells were transfected with a pool of siRNAs targeting Sik1,2 and 3 or scrambled control (siCtrl). 2 days post transfection cells were activated with TNFα (100 ng/μl) for 24 h. Viability was assessed by flow cytometry using an Aqua live-dead stain (mean + S.E.M., n=2-3).

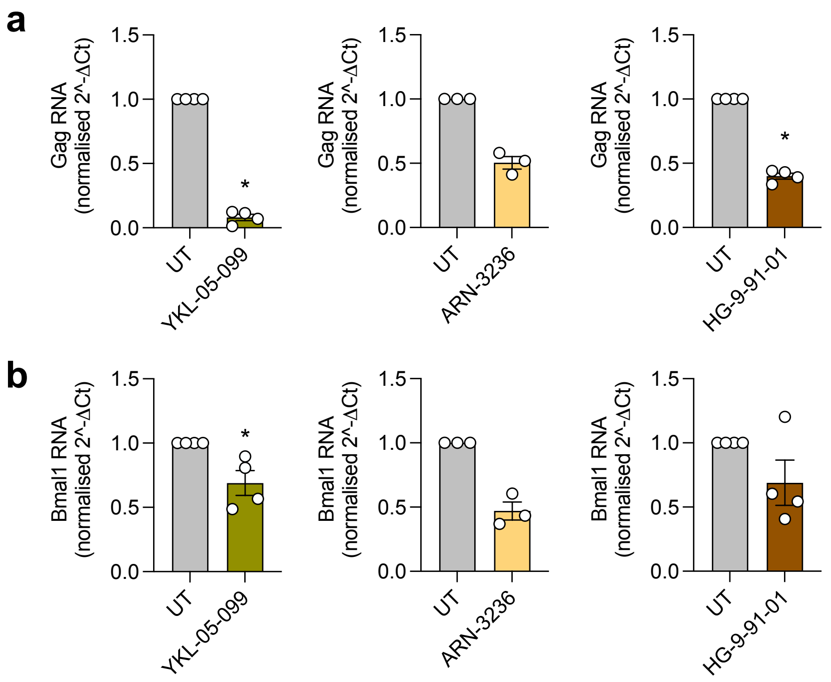

Supplementary Figure 3. Salt inducible kinase inhibitors reduce Gag and Bmal1 transcript levels in CD8 depleted PBMCs. CD8 depleted PBMCs were activated with IL-2, anti-CD3 and anti-CD28 for 3 days, cells were infected with HIV NL4.3-luc VSV-G for 24 h and treated with YKL-05-099 (2.5 μM), ARN-3236 (2.5 μM) or HG-9-91-01 (5 μM) for 24 h. Cells were lysed, RNA extracted and Gag **(a)** or Bmal1 **(b)** transcripts quantified by qPCR relative to B2M housekeeper (mean ± S.E.M., n=3-5, Mann-Whitney test). Data is shown relative to control untreated (UT) cells. The first two biological repeats employed cells from single donors, whilst repeats three and four employed cells pooled from three donors. * denotes $p<0.05$.

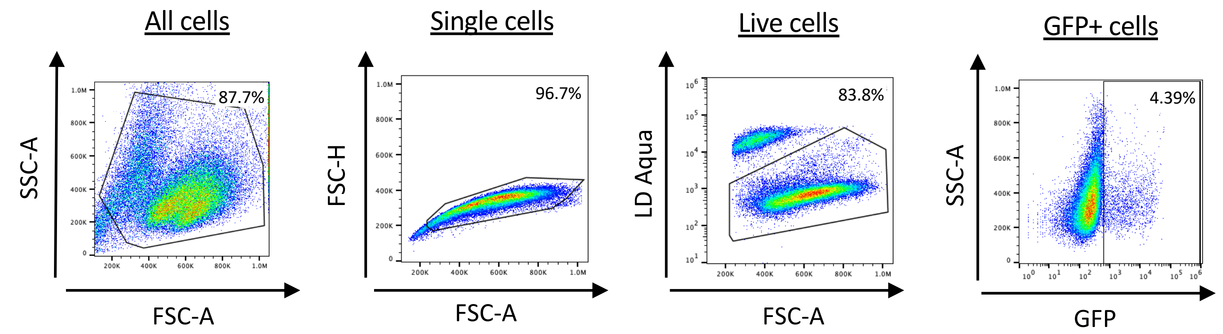

Supplementary Figure 4. Flow cytometry gating strategy. Representative dot plots illustrating gating strategy and analysis of J-Lat cells treated with 100 ng/µl TNFα. SSC = Side Scatter, FSC = Forward Scatter, LD = Live-Dead stain.
